# Long-Term Safety and Efficacy of Subcutaneous Cladribine Used in Increased Dosage in Patients with Relapsing Multiple Sclerosis: 20-Year Observational Study

**DOI:** 10.3390/jcm10215207

**Published:** 2021-11-08

**Authors:** Konrad Rejdak, Adriana Zasybska, Aleksandra Pietruczuk, Dariusz Baranowski, Sebastian Szklener, Magda Kaczmarek, Zbigniew Stelmasiak

**Affiliations:** Department of Neurology, Medical University of Lublin, 20-594 Lublin, Poland; mikusadriana@gmail.com (A.Z.); aleksandrapietruczuk@umlub.pl (A.P.); dariusz.baranowski@umlub.pl (D.B.); sebastian.szklener@umlub.pl (S.S.); mgdgasior@gmail.com (M.K.); zbigniewstelmasiak@umlub.pl (Z.S.)

**Keywords:** subcutaneous cladribine, relapsing multiple sclerosis, long-term efficacy, safety

## Abstract

Cladribine is currently registered as a 10-milligram tablet formulation with a fixed cumulative dosage of 3.5 mg/kg over 2 years. It is important to investigate if an increased dosage may lead to further clinical stability with preserved safety. This study used an off-label subcutaneous (s.c.) formulation of cladribine and compared outcomes (Expanded Disability Status Scale (EDSS) scores and disease progression) between 52 relapsing multiple sclerosis (RMS) patients receiving different s.c. dosing regimens with up to 20 years of follow-up. The study group received induction therapy with s.c. cladribine (1.8 mg/kg cumulative dose; consistent with 3.5 mg/kg of cladribine tablets). Patients were subsequently offered maintenance therapy (repeated courses of 0.3 mg/kg s.c. cladribine during 5–20-year follow-up). Forty-one patients received an increased cumulative dose (higher than the induction dose of 1.8 mg/kg); 11 received the standard induction dose. Risk of progression on the EDSS correlated with lower cumulative dose (*p* < 0.05) and more advanced disability at treatment initiation (*p* < 0.05) as assessed by EDSS change between year 1 and years 5 and 10 as the last follow-up. Maintenance treatment was safe and well-tolerated, based on limited source data. Subcutaneous cladribine with increased cumulative maintenance dosage was associated with disease stability and favorable safety over a prolonged period of follow-up (up to 20 years) in RMS patients.

## 1. Introduction

There has been substantial progress in understanding the pathogenesis of multiple sclerosis, which has led to the introduction of many successful disease-modifying drugs targeting different molecular pathways [1]. Among them, cladribine displays a unique mode of action [2]; it is a chlorinated analogue of deoxyadenosine that is relatively resistant to adenosine deaminase (ADA)-mediated deamination and accumulates in lymphocytes after it enters the cells. After intracellular activation by kinases, activated cladribine is cytotoxic to dividing and non-dividing lymphocytes due to its interference at several stages in nucleotide metabolism [3]. It has been extensively studied in different formulations [4,5,6], which finally led to the formal registration of a tablet formulation for the treatment of relapsing multiple sclerosis (RMS) [7,8]. The principle of its activity is referred to as induction therapy, consisting of pulsed selective cell depletion with secondary immune reconstitution affording long-lasting immune and clinical stabilization [9].

Cladribine is currently registered as 10-milligram tablets (MAVENCLAD^®^) with a fixed cumulative dose of 3.5 mg/kg body weight divided into two treatment cycles with a 1-year interval. In the CLARITY study, its efficacy was proven, with therapeutic effects lasting 3–4 years after treatment initiation [7]. Despite previous studies including higher doses of cladribine, there is no consensus on whether it is safe to use repeated courses of treatment with a higher cumulative dose than the approved treatment regimen (3.5 mg/kg) to retain long-term clinical efficacy. Thus, it is important to investigate if increased dosage of cladribine might lead to further clinical stability with preserved safety following long-term observation.

In the current study, we used an off-label subcutaneous (s.c.) formulation of cladribine, previously reported to be safe and efficacious [10,11]. Our aim was to compare clinical outcomes between patients receiving different dosing regimens of s.c. cladribine with up to 20 years of follow-up.

## 2. Materials and Methods

### 2.1. Study Population

This was a retrospective, observational study to assess the long-term efficacy and safety of s.c. cladribine in patients with RMS. The study design was approved by the local ethics committee. Patients treated in the Lublin MS center from 1996 to 2020 were considered for inclusion in the study.

Patients with clinically diagnosed RMS fulfilling the McDonald criteria [12] and receiving active treatment with off-label s.c. cladribine were invited for a final assessment conducted in 2020. We retrospectively analyzed the demographic and clinical data collected prior to the final assessment between the initiation of s.c. cladribine treatment and the final visit [10].

Exclusion criteria included acute relapse experienced within the preceding 1 month and refusal to participate. There were additional exclusion criteria in our MS center applied to patients considered for off-label treatment with s.c. cladribine. Concomitant diseases which, in the opinion of the attending physician, had a potential impact on the general status of patients and outcome prevented the patient from participating in the treatment program, such as active infectious diseases, neoplasm, and organ insufficiency (liver, kidney, heart, and pulmonary). In addition, patients who were immunocompromised (solid organ transplant or bone marrow transplantation) or had other diseases not mentioned but with a high risk of complications were not eligible for the study.

### 2.2. Treatments

Induction treatment: Cladribine was administered s.c. at a cumulative dose of 1.8 mg/kg (divided into 6 courses administered every 5 weeks for 4–6 days, depending on the patient’s body weight).

Maintenance treatment: All patients were offered the choice to continue maintenance treatment consisting of repeated courses of s.c. cladribine at an annual dose of 0.3 mg/kg, administered over 4–6 days, depending on patients’ body weight. Disease activity, progression, and patient choice dictated the length of maintenance therapy.

### 2.3. Assessments

Expanded Disability Status Scale (EDSS) scores were assessed for all patients at baseline, at the end of the s.c. cladribine induction treatment course (year 1), and during the observation period (years 5, 10, 15, and 20) depending on the duration of maintenance therapy. Disease progression was defined as a change in EDSS score of ≥0.5 between baseline and the last follow-up visit.

At the final visit, all patients had blood tests conducted to assess basic measures.

### 2.4. Data Analysis and Statistics

Baseline and follow-up data were compared using Fisher’s exact test, the unpaired t-test with the Welch correction, the Mann–Whitney test, and the Kruskal–Wallis test (non-parametric ANOVA) with Dunn’s multiple comparisons test, where appropriate. Two-sided tests were used throughout, and a *p*-value of <0.05 was considered statistically significant. Values are presented as mean ± standard deviation (SD).

Disease progression was analyzed in two separate approaches. Firstly, we assessed baseline EDSS score at treatment initiation with further follow-up of patients at specific time points: year 1 (induction treatment termination) and years 5, 10, 15, and 20 of follow-up. Estimates of time to EDSS progression (TTP) were calculated using the Kaplan–Meier method. The effects of four basic characteristics (gender, cladribine cumulative dose, age at treatment initiation, and disease duration to treatment initiation) on progression were examined using the univariate and multivariate Cox regression models. Gender was regarded as a categorical variable (female/male) whereas the other covariates were treated as continuous variables. The results of the Cox analyses are expressed as a hazard ratio with 95% confidence intervals (CI) and a *p*-value. In all analyses, a *p*-value of <0.05 was considered statistically significant. Secondly, we assessed EDSS score change between year 1 (induction treatment termination) and the subsequent time points of years 5, 10, 15, and 20.

GraphPad InStat 3.05 (GraphPad Software Inc., San Diego, CA, USA) was used for statistical analysis, while the time-to-event analysis was performed using JMP 15.2.0 Software (SAS Institute Inc., Cary, NC, USA).

## 3. Results

The study group consisted of 52 patients diagnosed with RMS who received induction therapy and were offered maintenance therapy over a 5–20-year observation period. Within the group, 41 patients received an increased cumulative dose of s.c. cladribine (higher than the induction dose of 1.8 mg/kg) while 11 only received the standard induction dose. Patients from the maintenance treatment subgroup received variable and higher cumulative doses, while those from the induction treatment subgroup all had only 5 years of follow-up. Thus, it was not possible to compare clinical characteristics between these two subgroups directly. We therefore summarized the demographics and clinical characteristics of the patients according to the different periods of follow-up (5, 10, 15, and 20 years), as presented in Table 1.

Subsequently, we performed an analysis of demographic and clinical characteristics based on the cumulative duration of follow-up, as summarized in Table 2.

### 3.1. Efficacy

#### Progression Analysis

**Figure 1:**
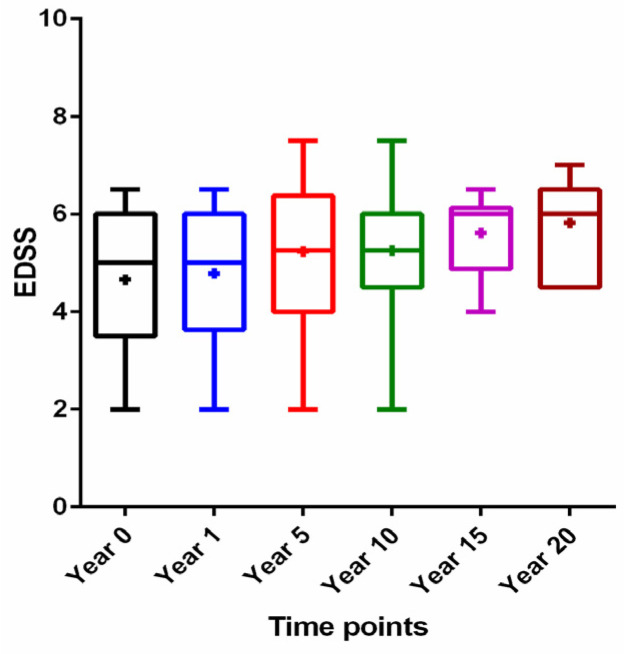
Expanded Disability Status Scale (EDSS) score over time: year 0 (at the cladribine induction treatment initiation), year 1 (at the end of cladribine induction treatment), year 5 (follow-up of 5 years), year 10 (follow-up of 10 years), year 15 (follow-up of 15 years), and year 20 (follow-up of 20 years). Data are presented as median (horizontal line), 25% interquartile range (box), range (whiskers), and means (middle cross).

As a first step, we analyzed the progression of disability in treated patients over the observation period, starting from baseline (at initiation of induction treatment) until the end of maximal follow-up. There was a gradual increase in EDSS score with overall statistical significance (*p* = 0.02) but without significant differences between selected subgroups (Figure 1).

**Figure 2:**
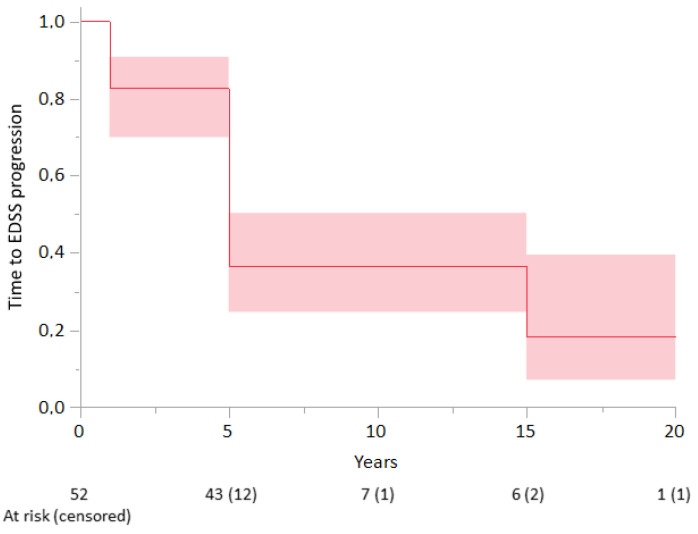
Time to Expanded Disability Status Scale (EDSS) score progression according to Kaplan–Meier analysis in the whole patient cohort (*n* = 52). The shading represents 95% confidence intervals.

Additionally, we analyzed the risk of the progression in correlation with basic demographic and clinical characteristics. Considering the period starting with treatment induction, the median time to EDSS score progression was 5 years, and the probability of being EDSS score progression-free was 37% (95% confidence interval (CI): 32–42%) at 5 and 10 years and 18% (95% CI: 16–21%) at 15 years (Figure 2).

No factors were identified as being significantly associated with neurological progression in both univariate and multivariate Cox analyses (*p* > 0.05) (Table 3). However, a one-unit (1 mg/kg) increase in cumulative cladribine dose was associated with a 7% reduction in the risk of progression (hazard ratio of 0.93 in multivariate analysis). Similarly, a trend for better outcomes was observed in women (47% hazard ratio increase in men compared to women). Nonetheless, the low number of patients within the subgroups limits the power to detect the variables significantly associated with the TTP outcome.

In the second step, we assessed EDSS score change between year 1 (induction treatment termination) and subsequent follow-up time points (years 5, 10, 15, and 20) in terms of correlation with baseline characteristics based on cumulative data. Table 4 demonstrates the baseline characteristics of progressive and non-progressive patients assessed at year 5. Higher cumulative s.c. cladribine dose was associated with better outcomes without clinical progression of EDSS score in RMS patients (*p* = 0.02). More advanced disability on the EDSS at baseline was also correlated with the risk of progression at year 5 (*p* = 0.03).

Similarly, Table 5 presents the baseline characteristics of progressive and non-progressive patients at year 10. Clinical progression was associated with a lower cumulative dose of s.c. cladribine (*p* = 0.01). Due to the small number of patients, these analyses were not performed at years 15 (progressive, *n* = 15; non-progressive, *n* = 3) and 20 (progressive, *n* = 10; non-progressive, *n* = 1).

### 3.2. Safety

Table 6 summarizes the medical events reported by patients at the final follow-up visits at 5, 10, 15, and 20 years after s.c. cladribine treatment initiation. Overall, these conditions seemed to be comorbidities related to multiple sclerosis and did not appear to be associated with s.c. cladribine treatment alone. It is noteworthy that none of the patients died or had severe events leading to treatment withdrawal. In two patients, the maintenance treatment was suspended in relation to medical status. In one case, a diagnosis of breast cancer was made 18 years after s.c. cladribine treatment initiation, during the maintenance phase of therapy (cumulative dose of 160 mg). The patient was diagnosed on routine screening and qualified for mastectomy, chemotherapy, and radiotherapy. Based on 7-year follow-up, there was no evidence of recurrence or metastasis, and her neurological status was stable. The second patient of special interest was diagnosed with pulmonary tuberculosis; it was not clear whether this resulted from reactivation of occult infection or new exposure. The patient received a cumulative dose of 180 mg of s.c. cladribine, with the diagnosis made during the maintenance phase of therapy. The diagnosis was made 6 years after the last dose of s.c. cladribine. The patient was treated with a full course of antibiotic treatment over 6 months and fully recovered from the infection.

In general, maintenance treatment with s.c. cladribine with an increased cumulative dosage was safe and well-tolerated.

## 4. Discussion

This is the first study to demonstrate long-term data on the efficacy and safety of off-label s.c. cladribine given in higher cumulative doses with up to 20 years of follow-up.

Briefly, s.c. cladribine was administered at an average dose of 1.8 mg/kg. This corresponds with the oral dose of 3.5 mg/kg used in a pivotal study of cladribine tablets (CLARITY) and is currently the registered dose for common practice [7]. It is well established that cladribine can be administered orally, s.c., or intravenously [13,14]. Considering that the bioavailability of cladribine, administered orally, is approximately 50%, in order to obtain a blood concentration and pharmacokinetic profile that mimics that of s.c. or intravenous administration, the dose had to be doubled [13]. Our s.c. dosing induction schedule differed from that of the CLARITY study by the duration of the treatment. In our study, the induction dose of s.c. cladribine was given within 6 months on average, divided into six equal doses, while in the CLARITY study, the oral dose of 3.5 mg/kg was given over 2 years as two short weekly treatments in each year [15]. In addition, the majority of patients in this study received maintenance doses over an extended time period, depending on their clinical status. In the current study, there was no control group to compare clinical efficacy. There was disease progression with a slow, gradual increase in mean EDSS score at consecutive time points of 5, 10, 15, and 20 years. However, patients from this cohort decided to continue treatment and were satisfied with its effects over a prolonged period of time. We found that the risk of progression correlated with lower cumulative drug dose and higher EDSS score at treatment initiation.

This study highlights the importance of safety assessment during treatment with cumulative dosing of s.c. cladribine as 80% of patients received a dose higher than 1.8 mg/kg, which is equivalent to the currently registered dose for common practice of 3.5 mg/kg of cladribine tablets. Variable maintenance treatment regimens were used, and administration of additional doses depended on the patients’ decision. We assessed the cumulative dose of s.c. cladribine for each patient over an observation period in order to assess adverse events. We reported several medical conditions diagnosed after treatment initiation, most of which can be regarded as comorbidities and not related to s.c. cladribine treatment [16]. In this cohort, only four patients had transient reversible lymphopenia of grade 2 at different time points of observation. Malignancies and infections were of special concern relating to cladribine treatment. There was one patient with diagnosed breast cancer who underwent mastectomy with a good outcome. We also reported one patient with tuberculosis, which was most likely related to cladribine treatment. This patient received full antibiotic treatment and recovered from the infection.

The results are consistent with previously reported safety data for cladribine tablet treatment [17,18]. Three categories of adverse events—namely lymphopenia, infections, and malignancies—were pre-defined as serious adverse effects of special interest (AESIs). With regard to malignancies, standardized incidence ratios (SIRs) were calculated in relation to the matched GLOBOCAN 2012 reference population [19]. The rate of malignancies observed with 3.5 mg/kg cladribine tablets in the final integrated safety analysis was not different from the expected rate in the matched GLOBOCAN reference population. There was no difference between the 3.5 mg/kg cladribine tablets group and the placebo in the overall incidence of infections. However, herpetic infections occurred more frequently in the 3.5 mg/kg cladribine tablets group (driven primarily by herpes zoster, followed by oral herpes and herpes simplex). Pulmonary tuberculosis was also reported among other infections. The long-term data for s.c. cladribine given in increased cumulative doses over a prolonged time period in the present study therefore support up-to-date observations for cladribine tablet treatment, suggesting that repeated courses of the drug can keep MS stable and are generally well tolerated by patients. The current study is consistent with previous reports that cladribine treatment exerts long-lasting clinical and immunological effects, including intrathecal oligoclonal band disappearance [10]. In view of previous and current reports, it seems that primary induction therapy with cladribine is crucial to achieve clinical stabilization, which could be maintained further by repeated doses over time. All of the patients were satisfied with the treatment effect in the present study and decided to continue treatment, having other drug options available. Further studies are, however, needed in order to establish the dosing schedule for repeated courses of cladribine after induction therapy within a registered dosing regimen.

There are several limitations of this study. It is a retrospective analysis of long-term outcomes of patients; thus, we had limited access to clinical and laboratory data. Similarly, we could not retrieve imaging data to correlate with the clinical status of our patients, which was assessed only by EDSS score. In particular, it would be interesting to calculate NEDA score (no evidence of disease activity) based on a multifactorial assessment as an additional measure. Regarding the monitoring of safety, patients had only routine assessments as part of their standard medical care, and we did not use a protocol-based monitoring program. This could lead to the omission of some medical events during observation periods. Further studies are needed to assess the efficacy and safety of currently registered cladribine tablets used with repeated dosing in order to maintain the therapeutic effect of induction.

## 5. Conclusions

The findings of this retrospective study indicate that treatment with s.c. cladribine with increased cumulative maintenance dosing was associated with disease stability and a favorable safety profile over a prolonged period of follow-up (up to 20 years) in patients with relapsing multiple sclerosis. Such findings warrant cautious interpretation in view of the study design and limited number of patients.

## Figures and Tables

**Table 1:** Baseline characteristics of patients according to duration of follow-up.

Baseline Characteristics	Total (*n* = 52)	5 Years (*n* = 26)	10 Years (*n* = 8)	15 Years (*n* = 7)	20 Years (*n* = 11)	*p*-Value
Gender (female/male)	37/15	18/8	6/2	5/2	8/3	>0.05
Age at MS onset, years (mean ± SD)	30.9 ± 8.2	32.2 ± 9.8	28.4 ± 5.4	31.5 ± 6.7	29.4 ± 6.1	>0.05
Age at treatment initiation, years (mean ± SD)	39.2 ± 8.6	44.2 ± 8.9	34.0 ± 3.8	33.5 ± 6.7	34.3 ± 4.6	>0.05
Cladribine (s.c.) cumulative dose at final follow-up (mg/kg body weight; mean ± SD)	2.6 ± 1.1	2.1 ± 0.4	2.7 ± 1.4	2.7 ± 1.3	3.8 ± 1.4	<0.001 *
EDSS score at treatment initiation (mean ± SD)	4.7 ± 1.4	5.5 ± 1.1	4.3 ± 1.6	3.9 ± 1.2	3.6 ± 1.0	<0.001 **
Prior treatment (*n* (%))	5 (10)					
Interferon beta-1a	1 (2)		1 (12)			
Interferon beta-1b	3 (6)	3 (12)				
Glatiramer acetate	1 (2)	1 (4)				
Comorbidities (*n* (%))	11 (21)					
Hypertension	2 (4)	2 (7)				
Dyslipidemia	2 (4)	1 (4)			1 (9)	
Paroxysmal atrial fibrillation	1 (2)	1 (4)			-	
Bradycardia (transient)	1 (2)				1 (9)	
Fatty liver disease	1 (2)				1 (9)	
Microcytic anemia	1 (2)	1 (4)				
Pulmonary emphysema	1 (2)		1 (12)			
Thyroid nodules	1 (2)	1 (4)				
Hypothyroidism	1 (2)		1 (12)			
Appendicitis (with appendectomy)	1 (2)				1 (9)	
Nephrolithiasis	1 (2)				1 (9)	

*n*, number of patients; MS, multiple sclerosis; s.c., subcutaneous; SD, standard deviation. * Overall difference in *p*-value, <0.001; year 5 vs. year 20, <0.001 (on multiple comparisons test). ** Overall difference in *p*-value, <0.001; year 5 vs. year 15, <0.05; year 5 vs. year 20, <0.001 (on multiple comparisons test).

**Table 2:** Baseline characteristics based on cumulative duration of follow-up.

Baseline Characteristics	5 Years (*n* = 52)	10 Years (*n* = 26)	15 Years (*n* = 18)	20 Years (*n* = 11)	*p*-Value
Gender (female/male)	37/15	19/7	13/5	8/3	>0.05
Cladribine (s.c.) cumulative dose at time point (mg/kg body weight; mean ± SD)	2.1 ± 0.4	2.5 ± 1.0	2.7 ± 1.1	3.8 ± 1.4	<0.001 *
Age at treatment initiation (mean ± SD)	39.5 ± 8.8	34.8 ± 5.9	35.2 ± 6.7	34.2 ± 4.6	0.02 **
Disease duration to treatment initiation (mean ± SD)	9.0 ± 8.6	5.5 ± 5.1	5.5 ± 5.4	5.1 ± 5.0	>0.05
EDSS score at treatment initiation (mean ± SD)	4.6 ± 1.4	3.8 ± 1.2	3.6 ± 1.0	3.4 ± 1.0	0.002 ***

EDSS, Expanded Disability Status Scale; *n*, number of patients; s.c., subcutaneous; SD, standard deviation. * Overall difference in *p*-value, <0.001; year 5 vs. year 10, <0.05; year 5 vs. year 20, <0.001. ** Overall difference in *p*-value, 0.02; no significant differences between selected time points. *** Overall *p*-value, 0.002; year 5 vs. year 15, <0.05; year 5 vs. year 20, <0.001. Kruskal–Wallis test (non-parametric ANOVA) followed by Dunn’s multiple comparisons test.

**Table 3:** Univariate and multivariate analyses of factors influencing time to EDSS score progression.

Variable	Univariate Cox Regression		Multivariate Cox Regression	
	HR (95% CI)	*p*-Value	HR (95% CI)	*p*-Value
Gender (male vs. female)	1.28 (0.62; 2.63)	0.5162	1.47 (0.69; 3.13)	0.3223
Disease duration to treatment initiation, years	0.97 (0.92; 1.01)	0.1428	0.96 (0.91; 1.02)	0.1585
Age at treatment initiation, years	0.99 (0.95; 1.03)	0.4702	1.00 (0.96; 1.05)	0.9584
Cumulative s.c. cladribine dose, per 1 mg/kg body weight	0.94 (0.69; 1.28)	0.6916	0.93 (0.68; 1.28)	0.6556

CI, confidence interval; HR, hazard ratio; s.c., subcutaneous.

**Table 4:** Baseline characteristics of progressive and non-progressive patients at year 5, based on analysis of change in EDSS score from year 1 (induction treatment termination).

Baseline Characteristics	Non-Progressive (*n* = 16)	Progressive (*n* = 36)	*p*-Value
Gender (female/male)	12/4	25/11	>0.05
Age at treatment initiation, years (mean ± SD)	42.8 ± 9.1	38.9 ± 8.4	>0.05
Disease duration to treatment initiation, years (mean ± SD)	10.1 ± 5.8	11.1 ± 6.0	>0.05
Cladribine (s.c.) cumulative dose at year 5 (mg/kg body weight; mean ± SD)	2.3 ± 0.6	1.9 ± 0.4	0.02
EDSS score at cladribine initiation (mean ± SD)	4.2 ± 1.2	5.5 ± 1.4	0.03

EDSS, Expanded Disability Status Scale; *n*, number of patients; s.c., subcutaneous; SD, standard deviation.

**Table 5:** Baseline characteristics of progressive and non-progressive patients at year 10, based on analysis of change in EDSS score from year 1 (induction treatment termination).

Baseline Characteristics	Non-Progressive (*n* = 5)	Progressive (*n* = 21)	*p*-Value
Gender (female/male)	5/0	14/7	>0.05
Age at treatment initiation, years (mean ± SD)	32.8 ± 2.6	35.3 ± 6.4	>0.05
Disease duration to treatment initiation, years (mean ± SD)	8.1 ± 3.8	9.1 ± 2.1	>0.05
Cladribine (s.c.) cumulative dose at year 10 (mg/kg body weight; mean ± SD)	3.2 ± 1.0	2.3 ± 1.0	0.01
EDSS score at cladribine initiation (mean ± SD)	4.2 ± 1.4	3.7 ± 1.2	0.05

EDSS, Expanded Disability Status Scale; *n*, number of patients; s.c., subcutaneous; SD, standard deviation.

**Table 6:** History of medical events reported at the final follow-up visit.

Medical History Diagnosed after s.c. Cladribine Treatment Initiation (*n* (%))	Year 5 (*n* = 15)	Year 10 (*n* = 8)	Year 15 (*n* = 7)	Year 20 (*n* = 11)
Appendicitis (with appendectomy)				1 (9.1)
Atrial fibrillation (paroxysmal)	1 (6.7)			
Bradycardia (transient)				1 (9.1)
Breast cancer		1 (12.5)		
Cataract	1 (6.7)			1 (9.1)
Cervical cancer (preinvasive, Grade 0)				1 (9.1)
Cholecystolithiasis	2 (13.3)		1 (14.3)	
Diabetes type 2	1 (6.7)		1 (14.3)	1 (9.1)
Dyslipidemia	3 (20.0)	1 (12.5)	1 (14.3)	3 (27.3)
Fatty liver disease (non-alcoholic)	1 (6.7)			2 (18.2)
Hypertension	5 (33.3)		1 (14.3)	2 (18.2)
Ischemic heart disease	1 (6.7)			
Kidney cysts	1 (6.7)			
Lumbosacral discogenic syndrome				2 (18.2)
Lymphopenia (prolonged >1 month, reversible)	1 (6.7)	1 (12.5)		2 (18.2)
Microcytic anemia	1 (6.7)	1 (12.5)		
Mood disorder		1 (12.5)		
Nephrolithiasis				2 (18.2)
Osteoporosis		1(12.5)		
Ovarian cyst				1 (9.1)
Peptic ulcer disease (with gastrectomy)			1 (14.3)	
Pulmonary emphysema			1 (14.3)	
Renal fibrosis				1 (9.1)
Thyroid disorders *	6 (40.0)	2 (25.0)		
Trigeminal neuralgia				1 (9.1)
Tuberculosis				1 (9.1)
Urinary tract infection (frequent)	2 (13.3)			
Uterine fibroids		1 (12.5)		1 (9.1)

*n*, number of patients, s.c.; subcutaneous. * Thyroid nodules: 1 (6.7%) at year 5; hyperthyroidism: 5 (33.3%) at year 5; hypothyroidism: 2 (25%) at year 10.

## Data Availability

The data used in this study are available from the corresponding author on reasonable request.
